# Smartphone-Assisted Digital Image-Based Optical Biosensor Array for Quantification of Interleukin-8 Using Antibody-Conjugated Gold Nanoparticles

**DOI:** 10.3390/mi17070789

**Published:** 2026-06-28

**Authors:** Akhil Chandrakanth Komaram, Yen-Ta Tseng, Chu-An Chan, Shau-Chun Wang, Chun-Jen Huang, Lai-Kwan Chau

**Affiliations:** 1 Department of Chemistry and Biochemistry, National Chung Cheng University, Chiayi 621302, Taiwan; akhilsnm7@gmail.com (A.C.K.); chanchuan881018@gmail.com (C.-A.C.); chescw@ccu.edu.tw (S.-C.W.); 2 Center for Nano Bio-Detection, National Chung Cheng University, Chiayi 621302, Taiwan; tsengyentaozzy@gmail.com; 3 Department of Chemical and Materials Engineering, National Central University, Taoyuan 320217, Taiwan; cjhuang@ncu.edu.tw

**Keywords:** colorimetric sensor, nanoparticle-linked immunosorbent assay, biosensor array, smartphone-assisted, digital image, gold nanoparticles, intrleukin-8

## Abstract

We developed a smartphone-assisted digital image-based optical biosensor array using a planar glass slide with sensor spots in a 2 × 5 array format for point-of-care multiplex detection of biomarkers. The detection is based on the integration of the capture antibody (Ab^C^)-functionalized sensor array with a detection antibody-conjugated gold nanoparticle bioconjugate (AuNP@Ab^D^) in the presence of interleukin-8 (IL8) to form a sandwich-type AuNP@Ab^D^–IL8–Ab^C^ nanocomplex on the sensing spot surface. Thus, the colorimetric detection method can be applied to the quantitative analysis of IL8, a clinically relevant pro-inflammatory and pro-angiogenic biomarker. The sensing strategy utilizes digital image-based analysis via ImageJ software (V 1.54 g; Java 1.8.0_345 [64 − bit], Windows 8) to quantify the colorimetric signals generated by the light absorbance of surface-bound gold nanoparticles in response to an IL8 droplet sample of merely 8 μL on the planar glass surface, achieving a low detection limit of 0.23 pg/mL (27 fM) and good reproducibility with a coefficient of variation of 0.95%. Validation using IL8-spiked serum at concentrations of 1 × 10^−9^ M and 1 × 10^−10^ M showed minimal matrix effects with a detection accuracy of 99.5% and 106.1%, respectively. Hence, this low-cost portable digital image-based plasmonic nanoparticle-linked immunosorbent assay serves as an alternative to traditional enzyme-linked immunosorbent assays.

## 1. Introduction

The human immune system quickly responds to infection by releasing cytokines and chemokines, which orchestrate the process of inflammation. These signaling proteins mediate inflammation by regulating immune cell recruitment, vascular permeability, and angiogenesis. Recently, they have also been found to play important roles in cancer [1,2,3,4]. Hence, these chemotactic signals often serve as important prognostic biomarkers and diagnostic markers for early detection as well as for monitoring of treatment responses of numerous diseases, including infections, cancer, chronic and systemic inflammatory disorders. Among them, interleukin-8 (IL8, also known as CXCL8) was originally described as an 8.4 kDa chemokine playing an important role in pro-inflammation [5,6]. Now, IL8 has also emerged as a potent pro-angiogenic factor and a biomarker for cancer progression and prognosis [2,3]. It is often overexpressed in the tumor microenvironment and directly promotes blood vessel formation, dissemination, and metastasis at tumor developing stages, often it shows strong correlation with microenvironmental changes which are related to disease severity [2,3,4,7].

Currently, both conventional and advanced biosensor diagnosis methods can be employed to quantitatively detect the IL8 concentration in clinical samples, allowing the assessment of inflammation and infection, through their disease-specific relevant cut-off values. It has been reported that IL8 causes inflammation in cancer patients to result in serum levels of >18 pg/mL in comparison with serum levels of ~13 pg/mL in healthy individuals [8]. Although traditional methods, especially enzyme-linked immunosorbent assay (ELISA), have often been employed in clinical analysis of IL8, ELISA tests are limited in their applicability for point-of-care (POC) applications as they generally require complicated operation procedures and long analysis time, as well as limited sensitivity, with a limit of detection (LOD) at roughly the 10 pg/mL level, which is near to the physiological basal clinical level of 5–10 pg/mL [9] and therefore challenging for early-stage detection of IL8. In addition, the complexity and dynamic nature of the human immune system require rapid analysis of a complex panel of multiple biomarkers to improve the accuracy and throughput of cancer diagnosis. While many current advanced biosensors have been developed to a point of sufficient sensitivity for cancer diagnosis, most of them still have their limitations in fulfilling the goal of rapid multiplex POC diagnostics. Moreover, multiplex assays may face challenges in inter-assay variability and reproducibility. Current demand is developing next-generation diagnosis methods to enable decentralized multiplex detection with high sensitivity and selectivity. Along this trend, nanotechnology-enabled array-based biosensing platforms are promising to overcome the shortcomings of conventional methods in sensitivity, analysis time, multiplexability, cost, and portability in order to meet the needs of point-of-care diagnostics.

Considering the various nanotechnology-enabled array-based biosensing approaches, nanoplasmonic colorimetric biosensing approach based on gold nanoparticles (AuNPs) was selected due to its unique optical properties originating from the localized surface plasmon resonance (LSPR) of AuNPs to yield a high extinction coefficient and strong visibility in the visible light spectrum, together with the ease of bioconjugation with biomolecules and exceptional colloidal stability [10,11]. Unlike traditional enzyme and fluorescent labels, AuNPs exhibit plasmonic absorption and scattering without the need for additional reagents; this generates robust visual colorimetric signals that can be easily detected by the naked eye or quantified using a digital camera. These advantages make AuNPs a smart choice of nanomaterial for achieving simple, rapid, cost-effective, portable, and visual analysis of biomarkers with an LOD below 1 pg/mL on cheap planar glass substrate, with solid potentiality for multiplex detection.

**Figure 1:**
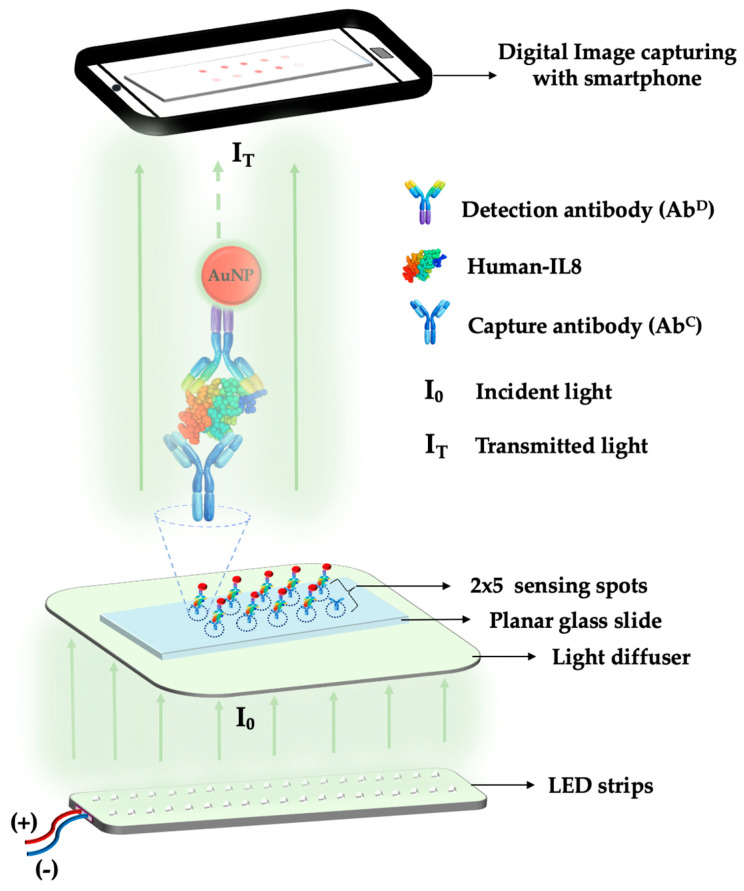
A schematic illustration of multiplex detection of the sandwich-type AuNP@Ab^D^–IL8–Ab^C^ nanocomplex formation on the surface of an array-based planar glass slide.

To develop a simple POC device on the basis of the optical response of an AuNP-labeled bioprobe in response to biomarker concentration variations on an array-based sensor plate, a simple and low-cost biosensing strategy as shown in Figure 1 was developed. This sensing approach is based on a sandwich immunoassay with a strong colorimetric signal exhibited by the plasmonic AuNP labels. After immobilizing a capture antibody on a sensing spot, the target IL8 molecules are selectively captured from a sample droplet. Subsequently, a AuNP-conjugated detection antibody binds to the captured IL8, forming a sandwich nanocomplex. Accumulation of AuNP labels on the sensing spot generates a concentration-dependent plasmonic colorimetric signal due to LSPR-induced absorption of visible light. Multiple sensing spots as an array on a sensor plate allows high-throughput detection of the same analyte in multiple samples or multiplex detection of multiple analytes in the same sample. Under controlled light-emitting diode (LED) light, the resulting transmitted light intensity is recorded using a smartphone camera. Then, it was quantified by ImageJ analysis by selecting the region of interest (ROI) of spots on the surface of the glass slide. The measured optical signal is inversely proportional to the amount of attached AuNPs and, consequently, to the concentration of IL8 in the sample. Integration of an IL8 biomarker detection strategy with this nanoplasmonic biosensing approach represents a promising way to get reliable and sensitive diagnosis results.

This paper reports on the development of a next-generation smartphone-assisted colorimetric [12] array-based biosensor that utilizes digital image analysis with user-friendly ImageJ software [13] for the quantification of the IL8 biomarker in buffer and spiked-serum samples. Smartphone-assisted setups could provide low-cost portable systems [14,15] and thus are an alternative to the conventional analytical devices. In this approach, the integration of the planar glass slide sensor array with a mobile phone to acquire a digital image enables portable and instrument-free detection. The digital image intensity value is directly correlated with the surface density of AuNPs in the nanocomplexes which is further proportional to IL8 concentration in a sample.

## 2. Experimental Section

### 2.1. Materials and Methods

Hydrogen tetrachloroaurate trihydrate, Tween 20, and hydrogen peroxide was purchased from Showa Chemical (Tokyo, Japan). N-Hydroxy-succinimide (NHS), 1-ethyl-3-(3-dimethylaminopropyl)-carbodiimine hydrochloride (EDC), and 2-[4-(2-hydroxyethyl)piperazin-1-yl]ethane-1-sulfonic acid (HEPES) were obtained from Thermo Fischer Scientific (Waltham, MA, USA). Trisodium citrate and sulfuric acid were provided by ITW Reagents (Glenview, IL, USA). Tris-base and magnesium sulfate were obtained from J.T.Baker (Radnor, PA, USA). Ethylalcohol and dimethyl sulfoxide (DMSO) were bought from HY Biocare Chem (Queens, NY, USA). 11-Aminoundecyltrethoxysilane (AUTES) was acquired from abcr GmbH (Karlsruhe, Germany). Disuccinimidyl suberate (DSS) was provided by Tokyo Chemical industry (Tokyo, Japan). 2-(N-morpholino) ethanesulfonic acid (MES) was supplied by Angene Chemical (Nanjing, China). Sulfobetaine silane (SBSi) [16] and silfobetaine thiol (SBSH) [17] were synthesized according to previously reported works. 16-Mercaptohexa decanoic acid (MHDA), bovine serum albumin (BSA), and human serum (product no. H4522) were purchased from Sigma-Aldrich (St. Louis, MO, USA). Anti-human IL8 capture antibody (Ab^C^, MAB2081), anti-human IL8 detection antibody (Ab^D^, MAB2082), and human IL8 (208-IL) standard were purchased from R&D Systems (Minneapolis, MN, USA). All aqueous reagents were prepared with pure water produced by a Milli-Q-water system (Merck Millipore, Darmstadt, Germany, 18.2 MΩ·cm resistivity). Microscope planar glass slides (76 mm × 26 mm × 1 mm) were purchased from Paul Marienfeld GmbH (Lauda-Konigshofen, Germany).

### 2.2. Synthesis of Detection Antibody-Conjugated Gold Nanoparticles

Citrate-capped gold nanoparticles (AuNPs) were synthesized by the trisodium citrate reduction (Turkevich) method following the previously reported procedures with minor changes [18,19]. In short, a 1% citrate solution was added quickly to a 0.88 mM chloroauric (III) acid (HAuCL_4_) solution under stirring at reflux temperature. The reaction was stopped after 20 min and the ruby-red color reaction mixture allowed to cool down at the room temperature. The concentration of the stock AuNP solution was adjusted by adding 0.12% of trisodium citrate solution until the peak absorbance value at about 519 nm was about 1 a.u. Citrate can play a dual role as a reducing and stabilizing agent to form nanoparticles without aggregation. The particle concentration of the stock AuNP solutions was calculated to be 4.11 nM, using a reported relationship of the extinction coefficient of gold nanoparticles with particle size [20].

**Figure 2:**
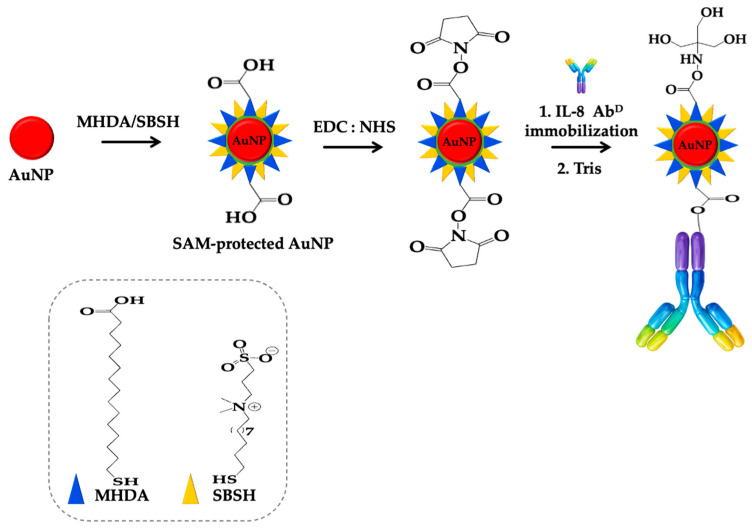
Schematic procedure of stepwise modification of AuNP surface: SAM binding site activation by EDC and NHS; formation of AuNP@Ab^D^ bioconjugate.

The AuNP surface modification procedure followed a previously described work [21] with minor changes, as shown in Figure 2. Aliquots of AuNP solution and pure water were mixed at a 1:1 (*v*/*v*) ratio, and a 2 mg/mL solution of Tween-20 was added to this mixture, then allowed to further mix for 1 h by shaking. Thereafter, a solution of MHDA and SBSH (0.5 mM each in ethanol, 1:9 *v*/*v*) was added to the above mixture to allow the formation of a mixed self-assembled monolayer (SAM) on the AuNP surface; thereupon, the resulting solution was incubated overnight at 13 °C by shaking. Afterward, the solution was centrifuged at 12,000× *g*, 15 °C for 25 min to remove the excess SAM precursors. The sediment containing the modified AuNPs was resuspended in an aqueous solution of 2 mg/mL Tween-20. The carboxylic groups on the SAM were activated by a 40 μL solution of EDC (0.026 M) and NHS (0.086 M) prepared in MES buffer (pH 5) added to a 1 mL AuNP solution. This mixture was incubated for 30 min at room temperature (R.T.) to form the NHS ester on the AuNP surface. The AuNP solution after activation was centrifuged for 25 min at 12,000× *g*, 15 °C, with the supernatant discarded and the sediment resuspended in HEPES (pH 7.5) buffer. Subsequently, an Ab^D^ solution of 2.4 × 10^−8^ M was added to the activated AuNP solution and incubated overnight at 13 °C in a shaking chamber to achieve the antibody immobilization. The Ab^D^-functionalized AuNP (AuNP@Ab^D^) solution was centrifuged at 10,000× *g* for 25 min, with the unreacted Ab^D^ in the supernatant removed and the bottom AuNP@Ab^D^ pellet resuspended in HEPES buffer, which contained 1 mM Tris-base and 2 mg/mL Tween-20 for deactivation of unreacted NHS esters groups on the AuNP surface. The deactivation step was carried out for 30 min under shaking at R.T. The centrifuge step as described above was repeated and the final sediment was resuspended in HEPES buffer containing 2 mM Mg^+2^. The concentration of the resulting AuNP@Ab^D^ solution was estimated to be 3.47 nM and used for subsequent detection experiments.

### 2.3. Functionalization of Sensing Spots with Anti-IL-8 Capture Antibody

**Figure 3:**
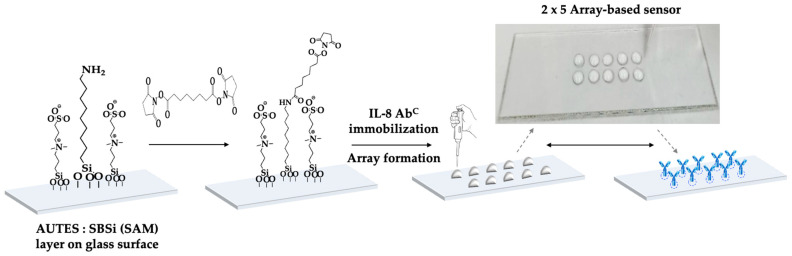
Schematic procedure of stepwise functionalization of 2 × 5 sensing spots on a planar glass slide: DSS crosslinked with binding site; Ab^C^ immobilized on glass surface. Upper right corner: a snapshot photograph during the surface functionalization of the 2 × 5 array-based biosensor.

The immobilization procedure of an anti-IL8 capture antibody on the glass surface of the sensing spots (Figure 3) was according to a reported procedure [21], with a few changes. A bare glass slide substrate was sonicated and washed with soap water, followed by immersion in piranha solution overnight. Thereafter, the glass slide was washed with pure water several times under sonication and dried at 70 °C overnight. The cleaned and dried glass slide was further cleaned and activated under oxygen plasma (PDC-001, Har-rick Plasma, 0.6 Torr, 30 W) treatment for 30 min. Thereafter, the glass slide was immersed in an ethanol solution containing AUTES and SBSi (3 mM each) and incubated overnight in a shaking chamber to form an SAM on the slide surface. The excess SAM precursors were rinsed off with ethanol. Subsequently, the SAM-coated glass slide was incubated with DMSO containing 80 mM DSS for 2 h under shaking to allow the formation of a covalent bond between the primary amine on AUTES and one of the NHS ester arms of the DSS crosslinker. Excess unreacted DSS crosslinker was rinsed off with ethanol and pure water. To functionalize each sensing spot, a droplet (8 μL) of Ab^C^ solution (1 × 10^−6^ M) in HEPES buffer (pH 7.5) was added to each sensing spot in a 2 × 5 array format. The modified glass slide with 2 × 5 sensing spots was incubated overnight at 13 °C to allow the amide bond conjugation of the Ab^C^ with NHS ester on another arm of DSS crosslinker. Following incubation, the glass slide was washed with buffer to remove unconjugated Ab^C^; thereupon, the remaining unreacted NHS ester groups were deactivated for 30 min by using 100 mM Tris and 1% BSA prepared in HEPES buffer. Each sensing spot was then ready for subsequent sensing experiments.

### 2.4. Conducting IL8 Detection Experiment

Individual IL8 standard solutions with concentrations ranging from 1 × 10^−8^ M to 1 × 10^−13^ M were prepared in HEPES binding buffer with 2 mM Mg^+2^. Each standard solution was subsequently added as a droplet (8 μL) onto a separate sensing spot to allow binding between Ab^C^ and IL8, while one spot did not have the IL8 standard added and served as a blank in order to assess the degree of background non-specific absorption. To understand the sensor responses due to matrix effects, two 10% diluted human serum samples were separately spiked with a 1 × 10^−9^ M or 1 × 10^−10^ M IL8 solution and added separately as a droplet (8 μL) on the designated sensing spots for 8 min incubation at R.T. The unbounded IL8 molecules were washed out with buffer. To detect any possible Ab^C^-IL8 complex formed on each sensing spot, the designated spots were incubated with an 8 μL droplet of AuNP@Ab^D^ solution for 8 min at R.T. to form the AuNP@Ab^D^–IL8–Ab^C^ nanocomplex structure (Figure 1). Finally, the array-based glass slide with sensing spots was washed with a buffer solution to clear the unbound AuNP@Ab^D^, and later on washed with pure water to remove salts and other residues on the sensing spots. The resulting sensing spots with bound AuNPs exhibited a light pink color and the 2 × 5 sensing spots on the glass slide were then used for colorimetric optical analysis.

### 2.5. A Portable Colorimetric Optical Detection Setup and Detection Principle

**Figure 4:**
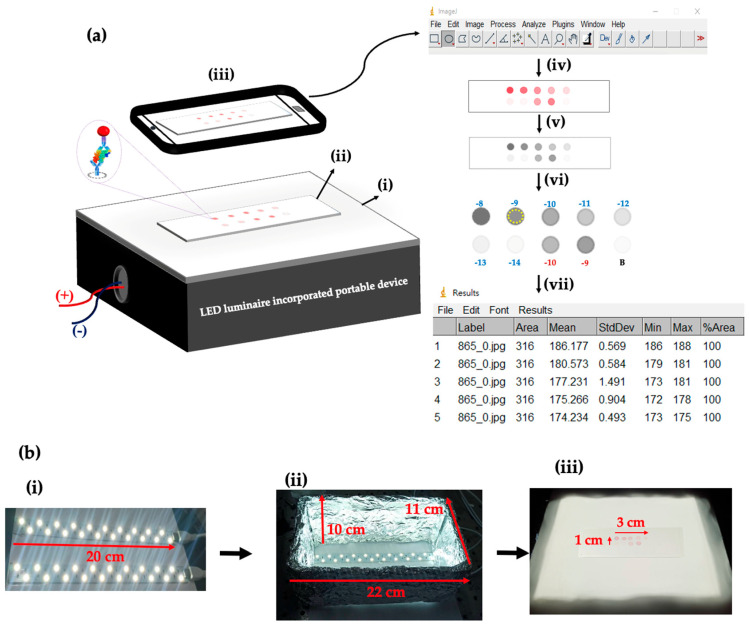
(**a**) Schematic illustration of the colorimetric detection setup and stepwise process of data extraction: (i) LED enclosure box; (ii) a glass slide containing an array of sensing spots, where each spot may have a different surface density of surface-bound AuNPs, is placed on the top of a white acrylic transparent sheet; (iii) acquiring the digital image by a smartphone in dark condition; (iv) uploading the acquired RGB digital image into ImageJ software; (v) conversion of RGB digital image into grayscale image; (vi) manually selecting a circular region (dashed yellow circle) in each sensing spot; (vii) extraction of the mean grayscale data. (**b**) photographic illustration of the light source setup and acquired images: (i) white LED strips; (ii) LED strips incorporated in the enclosure box; (iii) acquired images of a glass slide containing an array of sensing spots after sensing reactions, placed on top of the enclosure box sealed with a white acrylic sheet.

A portable colorimetric optical detection system as shown in Figure 4a has been developed specifically to provide uniform light illumination to an array-based glass slide which contained 10 sensing spots, where each sensing spot had been pre-functionalized with an anti-IL8 capture antibody (Ab^C^). In the presence of both IL8 and AuNP-labeled anti-IL8 detection antibody (AuNP@Ab^D^), a number of AuNP@Ab^D^–IL8–Ab^C^ nanocomplexes will be formed on a sensing spot, enabling digital image acquisition via a mobile phone. The device consists of a compact sealed enclosure connected to two parallel white LED strips (table lamp grade, Jin Hua Electronics) as the light source mounted on its bottom. The table lamp-based white LED strips were embedded in a small cotton box covered with aluminum foil except at the top. The top of this box was covered with a white acrylic sheet that acts as a translucent light diffuser, spreading the light evenly on the top of the box surface to ensure uniform distribution of light across all the sensing spots as well as to reduce heterogeneity (non-uniform illumination) and shadows, as shown in Figure 4b.

In the process of placing a dry flat glass slide containing an array of sensing spots that may have a various surface density of surface-bound AuNPs due to different applied IL8 concentrations, the light from the LED source across the light diffuser panel will produce an incident light (I_0_) with almost uniform intensity to transmit through the array of sensing spots. When the sensing spot contains surface-bound AuNPs, light absorption due to LSPR will occur, resulting in a decrease in the transmitted intensity (I_T_). As a result, this sensing spot will appear as a darker spot as compared to the background. This setup allows high-resolution digital images of the flat glass-slide sensor array after sensing reactions to be captured using a standard smartphone at a certain distance above the glass slide in dark conditions as shown in Figure 4a. Quantitative data related to the array of sensing spots were acquired using mobile phone hardware, and hence the data were extracted by using an open-source software ImageJ (V 1.54 g; Java 1.8.0_345 [64 − bit], Windows 8), as shown in steps iii to vii in Figure 4a. In other words, the mobile-phone-acquired RGB image can be converted into grayscale and then the data can be manually extracted from an individually selected circular region in each sensing spot in the array. Each obtained data point is calculated in the form of normalized transmitted intensity ∆I/I_0_, where ΔI = (I_0_ − I_T_), I_0_ represents the average transmitted light intensity through the unmodified glass surface areas (*n* = 3), and I_T_ represents the transmitted light intensity through the selected circular region in each sensing spot. The normalized transmitted intensity change corresponds to the optical response generated by the number of surface-bound AuNP@Ab^D^–IL8–Ab^C^ nanocomplexes in each sensing spot. The ROI was defined as an 8 μL droplet confined simply by surface hydrophobic interactions.

## 3. Results and Discussion

### 3.1. Characterization of AuNP@Ab^D^ and Its Binding with IL8

**Figure 5:**
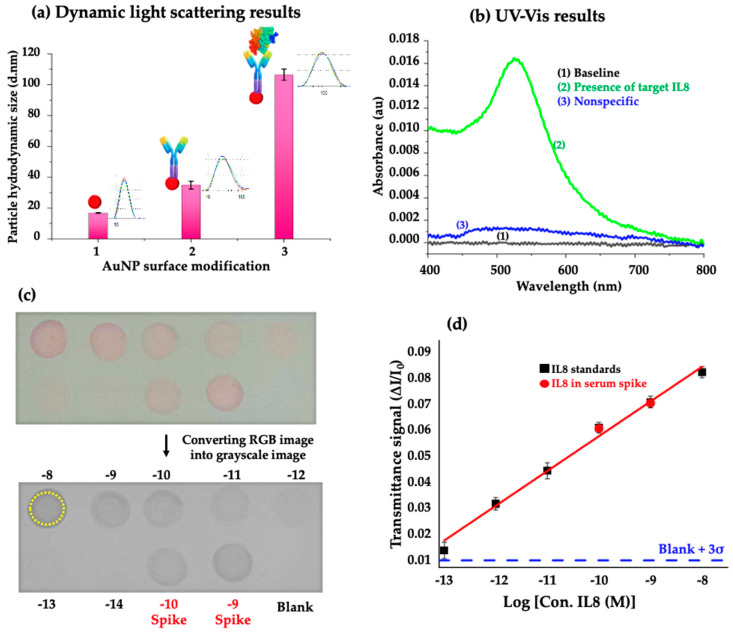
Characterization of AuNPs at different stages: (**a**) Average hydrodynamic diameter of AuNP measured at different reaction steps: 1—well-dispersed citrate-capped AuNPs; 2—after conjugation of AuNP with Ab^D^; 3—after binding of AuNP@Ab^D^ with IL8. (**b**) UV–Vis results of planar glass slide surface modification; bare glass slide baseline signal (Line 1, black); presence of IL8 target, AuNP@Ab^D^-IL8-Ab^C^ sandwich nanocomplex a gave strong absorption peak (Line 2, green); non-specific absorption signal (Line 3, blue) in the absence of IL8 target. (**c**) Smartphone-acquired digital RGB image of the planar glass slide sensor array after biosensing reaction, then converted into grayscale by ImageJ software. The sensing spots marked 1–10 correspond to the reaction of the capture probe in each sensing spot in the presence of AuNP@Ab^D^ and IL8 at concentrations of (1) 1 × 10^−8^ M, (2) 1 × 10^−9^ M, (3) 1 × 10^−10^ M, (4) 1 × 10^−11^ M, (5) 1 × 10^−12^ M, (6) 1 × 10^−13^ M, (7) 1 × 10^−14^ M, (8) 1 × 10^−9^ M IL8-spiked serum, (9) 1 × 10^−10^ M IL8-spiked serum, and (10) blank, where the dashed yellow circle represents the area selected for ImageJ analysis. (**d**) A calibration curve plotted with normalized intensity ΔI/I_0_ versus IL8 concentration. The black squares represent the data points using IL8 standards, red circle dots represent the results from IL8-spiked serum samples, and the red line represents the regression line. This average data were obtained from three different glass slide sensor arrays.

The successful conjugation of AuNPs with anti-IL8 detection antibody to form AuNP@Ab^D^ and then the binding of AuNP@Ab^D^ with IL8 to form a nanocomplex of AuNP@Ab^D^–IL8 was confirmed by measuring the hydrodynamic diameters of AuNPs at each stage using a dynamic light scattering (DLS) instrument (Malvern, Zetasizer Nano ZS90). Figure 5a illustrates that the average hydrodynamic diameter (D_h_) of citrate-capped AuNPs was 16.8 nm. After chemical conjugation with anti-IL8 detection antibody, the D_h_ of the nanoparticles increased to 36.9 nm, indicating successful conjugation of IL8 with citrate-capped AuNPs to form AuNP@Ab^D^. After further reaction of AuNP@Ab^D^ with IL8 at 1 × 10^−8^ M, the D_h_ of the nanoparticles further increased to 106.5 nm, suggesting that IL8 interacts with the anti-IL8 antibody on AuNP@Ab^D^ to form the AuNP@Ab^D^–IL8 nanocomplex.

### 3.2. Conformation of AuNP@Ab^D^–IL8–Ab^C^ Nanocomplex Formation on Glass Slide

The successful formation of AuNP@Ab^D^–IL8–Ab^C^ on a glass slide pre-functionalized with Ab^C^ was validated using UV–Vis spectroscopy (JASCO V-570) as illustrated in Figure 5b. The background absorption spectrum of a cleaned glass slide (Line 1, black) which exhibited minimal absorbance in the visible region was first measured. In the presence of IL8 at 10 nM and AuNP@Ab^D^ at 3.47 nM, the absorption spectrum exhibited a strong peak absorbance signal at 525 nm, 0.01647 a.u (Line 2, green), indicating the binding between Ab^C^ and the AuNP@Ab^D^-IL8 nanocomplex results in the formation of the sandwich-type AuNP@Ab^D^-IL8-Ab^C^ nanocomplex on the glass slide surface. On the other hand, in the presence of AuNP@Ab^D^ at 3.47 nM but in the absence of IL8, the absorption spectrum exhibited a very small peak absorbance signal at 525 nm (Line 3, blue), suggesting a small amount of AuNP@Ab^D^ remained on the glass slide surface even after washing with HEPES buffer. This phenomenon is attributed to the non-specific adsorption of AuNP@Ab^D^ on the sensor surface. Although the background signal due to non-specific adsorption of AuNP@Ab^D^ is small, it will cause misinterpretation of the signal as a positive signal. Therefore, a more effective anti-fouling surface will minimize the background signal. Practically, to eliminate the possibility of false-positive signals, in the next section we employed a cutoff value by calculating the mean plus three times the standard deviation of the measured background signal from samples of AuNP@Ab^D^ without IL8. In summary, the above UV–Vis spectra conform to the proposed plasmonic nanoparticle-linked immunosorbent assay (PNLISA) detection strategy for IL8 using AuNP@Ab^D^ to form a specific sandwich-type nanocomplex on glass slides is feasible.

### 3.3. Detection of IL8

The development of PNLISA from a single plex to a simultaneous multiplex detection format could create a revolution in diagnosis. This study employed IL8 as a model target to demonstrate the feasibility of the extension of the PNLISA method to the simultaneous multiplex detection strategy. In this approach, after a sensing reaction, high-resolution digital image data of a planar glass slide with a sensor array were first acquired using a standard smartphone as shown in Figure 5c. In the absence of IL8, the blank spot exhibited a negligible color, conforming minimal background non-specific binding (BNSB) between AuNP@Ab^D^ and Ab^C^ on the surface. This is attributed to the efficient BSA-Tris blocking step, highlighting the binding specificity in this PNLISA method. To avoid false positive sensor responses due to BNSB, we defined a threshold value as the average signal from a blank + 3σ, where σ is a standard deviation of the signal from the blank. In this experiment as shown in Figure 5d, the threshold value is 0.01014 (shown as horizontal dashed blue line). In addition, the glass slide sensor array has demonstrated its capability of visual quantitation wherein different color intensities develop at different IL8 concentrations. When the incident light transmits through the sensing spots, the surface-bound AuNPs strongly absorb light due to LSPR, resulting in color changes. At high IL8 concentrations, increasing nanoparticle density increases light absorption; in contrast, at low IL8 concentrations, decreasing nanoparticle density leads to lower absorption and hence higher light transmission was observed. After acquisition of the digital RGB image by a mobile phone, conversion of the digital RGB image into grayscale format can simplify the quantitative analysis due to the pixel intensity range from black to white only.

Repeated incubation and washing steps can cause uneven droplet spreading and color distribution, potentially increasing the dimensions of the wetted spots compared to the initial state; however, the use of consistent sample volumes, calibrated pipetting, and standardized washing procedures minimizes these variations, ensuring minimal effects on IL8 signal quantification. Non-uniformity in AuNP distribution was observed only at the periphery of the expanded wetted spots—possibly caused by simple aggregation of AuNP@Ab^D^ due to the coffee ring effect [22,23] as opposed to specific binding—and this region was therefore excluded from IL8 signal quantification. It should be noted that the coffee ring effect is minor as the droplet has almost the same volume in the whole incubation period. The analytical response primarily reflected the concentration-dependent accumulation of a uniformly distributed layer of AuNP@Ab^D^–IL8–Ab^C^ nanocomplexes within the hydrophilic sensing spot, enabling reliable and reproducible quantitative signal acquisition through ROI signal normalization. Future designs incorporating hydrophilic/hydrophobic boundaries or sample-containing micro-wells could further reduce wetting-related variability and enhance the assay’s robustness.

As shown in Figure 5d, a calibration curve plotting normalized transmitted light intensity versus IL8 concentration at all points (black square) shows a linear regression line (red line) with a correlation coefficient of R^2^ = 0.995. An LOD of 27 fM (0.23 pg/mL) was calculated from the calibration curve using its intercept k and slope m of 0.19217 and 0.01342, respectively, according to the following equations:

Y = mx + k,(1)

LOD = 10^X(LOD)^, (2)

where X(LOD) is the value obtained from Equation (1) when Y is the minimal distinguishable signal, i.e., when Y equals the threshold value of 0.01014 in this experiment.

To explore the feasibility of this glass slide sensor array for real biological sample detection, IL8 detection experiment was demonstrated with IL8-spiked serum samples to understand the matrix effects in real samples and to assess the applicability of the proposed method in real situations. In this investigation, the results of the detection assays of two known IL8 standards with concentrations of 1 × 10^−9^ M and 1 × 10^−10^ M were spiked into samples of 10% diluted human serum in HEPES buffer as test samples. As shown in Figure 5d, the results from the IL8-spiked samples closely matched the expected results predicted by the calibration curve, with detection accuracy values of 99.5% and 106.1% at 1 × 10^−9^ M and 1 × 10^−10^ M, respectively. These results indicate that the serum matrix has negligible interference along with IL8 detection and the planar glass slide sensor array has the potential to perform clinical sample analysis within 20 min.

### 3.4. Comparison with Other Detection Methods

Several immunoassay methods were developed for IL8 detection due to the important role of IL8 as a pro-inflammatory and pro-angiogenic biomarker in humans. The low LOD and wide linear detection range as well as the desirable features for POC applications presented in our work are impressive when compared to other current analytical techniques, as listed in Table 1. Compared to previously reported immunoassay and biosensing methods, the proposed nanoplasmonic colorimetric sensor array demonstrated highly competitive analytical performance for IL8 detection. The current method offers additional practical advantages—including a simple optical/colorimetric sensing mechanism and multiplex detection capability—that are highly suitable for POC applications. Overall, the key outcome of this work is the successful achievement of practical portability, characterized by highly sensitive IL8 detection, rapid assays, multiplex sensing capability, and instrument-free analysis. These advantages hold great promise for applications in clinical biomarker monitoring and rapid inflammatory disease screening.

## 4. Conclusions

In this study, we developed a simple, flexible, and fast-response array-based sensing platform for ultra-sensitive detection of the IL8 biomarker. The proposed optical biosensing system integrates AuNP-mediated colorimetric sensing on a planar glass surface, smartphone-assisted digital image acquisition, and low-cost quantitative analysis using ImageJ. This array-based sensing approach significantly improved the ability to simultaneously analyze multiple samples at different sensing spots in a single experiment, thereby reducing testing time compared to traditional hierarchical sensing approaches. IL8 was successfully detected in serum with high detection accuracy by this platform, which is of great importance for analysis of clinically important biomarkers in the future.

This promising detection strategy could enable multiplex biomarker detection through array integration in the future. In addition, the analytical accuracy, automation, and high-throughput capability can be further improved by upgrading the quantitative analysis with artificial intelligence-assisted automatic sensing region selection and signal extraction. Overall, this current research demonstrates a robust, scalable, and cost-effective biosensing approach for applications such as early disease diagnosis, inflammation monitoring, and cancer prognosis. Future studies will focus on improving multiplexing efficiency, clinical validation using patient samples, enabling machine learning and image analysis in a user-friendly manner, and optimizing the portable diagnostic device design for broader use in healthcare applications.

## Figures and Tables

**Table 1:** Comparison of immunoassay methods for IL8 and their LODs.

Method	Key Feature	Time	LOD	Ref
ELISA	Commercially available	90 min	1.8 pg/mL	[24]
SPR immunosensor	Label-free and real-time analysis	13 min	20 pg/mL (2.5 pM)	[25]
Bragg reflection based on resonant nanopillar	Label-free continuous monitoring	---	22.7 ng/mL (2.7 nM)	[26]
Impedance	Label-free detection	15 min	(10 fM)	[27]
Amperometric	Integrated microfluidic chip	---	0.1 pg/mL	[28]
Surface-enhanced Raman scattering	Spectral acquisition	>40 min	6.04 pg/mL	[29]
Nanoplasmonic colorimetric sensor array	Multiplex detection	20 min	0.23 pg/mL (27 fM)	Present work

## Data Availability

The raw data supporting the findings of this study are available from the corresponding author upon reasonable request.
